# Real-world Review of Adherence to Guideline-directed Intravenous Iron Therapy in Heart Failure Patients: A Retrospective Observational Study from a Tertiary Care Hospital in Oman

**DOI:** 10.2174/011573403X393791251014065153

**Published:** 2025-10-23

**Authors:** Muhammad Shoaib, Khamis Al Hashmi, Abdullah Al Alawi, Salim Al Busaidi, Said Al Jaadi, Yousef Al Shaidi, Ammar Al Naamani, Maryam Alsaidi, Abdullah Al Jabri, Yahya Al Attraqchi, Ahmed Abdelmoaty, Khalfan Al Zeedy, Adil Al Riyami

**Affiliations:** 1 Department of Medicine, Sultan Qaboos University Hospital, UMC, Muscat, Oman;; 2 Department of Physiology, College of Medicine and Health Sciences, Sultan Qaboos University, Muscat, Oman

**Keywords:** Iron deficiency, heart failure, intravenous iron therapy, ferric carboxymaltose, guideline-directed medical therapy, transferrin saturation

## Abstract

**Introduction:**

International guidelines recommend intravenous (IV) iron replacement in patients with heart failure (HF) with reduced or mildly reduced ejection fraction (HFrEF or HFmrEF) and iron deficiency (ID). IV iron therapy remains underutilised despite growing evidence of its positive impact on hospitalisation rates, quality of life, and symptom control in HF. In this study, we aimed to explore the prevalence of ID and adherence to guideline-directed medical therapy (GDMT) for ID in HF patients.

**Methods:**

It was a retrospective observational study performed at a tertiary hospital in Oman. All HF patients admitted between March 2022 and February 2024 were included. ID was defined as serum ferritin less than 100 µg/L or serum ferritin 100–299 µg/L with transferrin saturation (TSAT) of less than 20%. Patients in intensive care units, pregnant women, and those with HF with preserved LVEF (HFpEF) were excluded.

**Results and Discussion:**

Only 26% (n=97) of 376 patients with HFrEF/HFmrEF were screened for ID, and about half of them (n=52) were found to be iron deficient. ID was tested more often in patients with anemia compared to those without anemia (33.9% *vs*. 10.7%, *p* < 0.001). IV iron in the form of iron carboxymaltose (FCM) was administered in 19% of ID patients, but only 15% received the recommended dose of 1000 mg FCM. There was no statistically significant difference in patient demographics or comorbidities between patients with or without ID.

**Conclusion:**

Among 376 HF patients screened for ID, almost half were iron deficient. However, the compliance rate of IV FCM therapy remained quite low. Low screening rates and limited adherence to GDMT underscore the need for standardized hospital protocols for the management of ID in HF patients.

## 1 INTRODUCTION

Iron deficiency (ID) is one of the most common environmentally acquired nutritional deficiencies worldwide [1, 2]. Among patients with heart failure (HF), the prevalence of ID has been reported to be around 50% in chronic HF (CHF) and up to 80% in acute decompensated HF [3, 4]. Similarly, the prevalence of anemia increases from 35% in CHF to 50% in decompensated HF patients admitted to hospitals [5-7]. ID is usually defined as a serum ferritin level of less than 100 µg/L or between 100–300 µg/L with transferrin saturation (TSAT) of less than 20% [8]. This definition is used in major HF trials, such as FAIR-HF [9], and in the European Society of Cardiology (ESC) and American Heart Association (AHA) guidelines [10, 11]. However, other studies have used serum iron levels of less than 13 µmol/L as a definition of ID [12]. A study by Grote *et al*. showed a strong correlation between low serum iron levels and depleted bone marrow reserves in HF patients [13]. This discrepancy in various definitions of ID leads to variation in reported long-term outcomes of ID treatment in HF patients [5, 14].

ID has been associated with adverse prognosis, poor quality of life, and a decline in exercise capacity in patients with HF [15-17]. When coupled with anemia, it can also lead to increased cardiovascular mortality and hospitalisation rates [3, 18-20]. The pathophysiological link between anemia and ID in HF is complex, and it remains unclear whether these two factors are the result or the cause of HF [5, 21]. Iron replacement in HF with reduced ejection fraction (HFrEF) shows long-term benefits regardless of the presence or absence of anemia [9]. This finding has been validated in multiple randomized controlled trials and meta-analyses such as CONFIRM-HF (symptomatic HF patients with EF ≤45%), AFFIRM-AHF (HF with EF <50%), and the study published by Kapoor *et al*. in 2015 [22-24].

Oral iron replacement is not effective in improving HF-related morbidity or HF-related quality of life [25, 26]. The reduced efficacy of oral supplementation is likely secondary to poor compliance due to side effects, poor oral absorption, and inadequate dosing [14]. In contrast, intravenous (IV) therapy is more cost-effective and provides better short- and long-term benefits [27]. Therefore, both the AHA and ESC guidelines recommend IV iron replacement with ferric carboxymaltose (FCM) in all patients with HFrEF and HFmrEF with evidence of ID, with or without anemia [10, 11].

Despite the high prevalence of ID and strong evidence of the benefits of IV iron replacement, ID continues to remain underdiagnosed and undertreated in HF patients [4]. There are currently no published data in the Middle East regarding the compliance rate and barriers to providing IV iron according to recommended guideline-directed medical therapy (GDMT) for HF.

Aim and objectives: In this study, we aimed to investigate the prevalence of ID and anemia in HF patients admitted to this hospital. The secondary aim of this study was to assess adherence to the international guidelines on ID treatment in HF patients compared to the standard of care defined by the AHA guidelines.

## 2 MATERIALS AND METHODS

### 2.1 Study Design and Setting

This was a retrospective study performed at Sultan Qaboos University Hospital (SQUH), a tertiary care center in Oman, between 2022 and 2024. Patients admitted to any medical ward during the 24-month period were screened for inclusion and exclusion criteria. The data of selected patients were retrieved from the electronic database of the hospital using TraK Care software.

ID was diagnosed according to the FAIR-HF trial definition used by the ESC and AHA guidelines: serum ferritin of less than 100 µg/L or transferrin saturation of less than 20% in case of ferritin between 100–300 µg/L [10, 11, 28]. Anemia was defined as serum haemoglobin concentration of less than 13 g/dL in males and 12 g/dL in non-pregnant females [29, 30]. In this study, we used 1000 mg IV FCM as the standard of care for treatment of ID in HF, as per AHA guidelines [11].

### 2.2 Inclusion and Exclusion Criteria

All adult inpatients admitted under acute medicine or any medical specialty with echocardiographic evidence of HFrEF (EF < 40%) or HFmrEF (EF 41–50%) were included, while patients admitted to an intensive care unit, those with HF with preserved EF (HFpEF), and pregnant women were excluded.

### 2.3 Variables of Interest

The following variables were retrieved from the electronic database of the hospital using Trak Care software: patient demographics (age and gender); biochemical markers, including hemoglobin (Hb), mean corpuscular volume (MCV), mean corpuscular hemoglobin (MCH), serum creatinine, B-type natriuretic peptide (BNP), C-reactive protein (CRP), serum ferritin, serum iron, and TSAT; presence of chronic illnesses such as chronic kidney disease (CKD), ischemic heart disease (IHD), anemia, diabetes, smoking, peripheral vascular disease (PVD), and stroke; recent echocardiogram date and left ventricular ejection fraction (LVEF); and evidence of iron replacement during the admission, including the dose, biochemical type, and route of administration.

### 2.4 Statistical Analysis

Categorical variables were expressed as percentages, while numerical data were presented in the form of mean and median with interquartile range and frequency distribution curves. For non-normally distributed data, the Kruskal-Wallis test and chi-square tests were used to compare different groups.

### 2.5 Ethics

The ethics approval for this study was obtained from the Medical Research and Ethics Committee (MREC) of the College of Medicine and Health Sciences, Sultan Qaboos University (MREC #3243).

The research procedures performed in this study were conducted in accordance with the ethical standards of the Medical Research and Ethics Committee (MREC) of the College of Medicine and Health Sciences, Sultan Qaboos University, and with the principles of the Declaration of Helsinki (1975), as revised in 2013.

## 3 RESULTS

A total of 780 patients with HF were admitted to SQUH between March 1, 2022, and February 29, 2024. Out of these, 404 patients (51.8%) had a left ventricular EF greater than 50%, classifying them as HFpEF. The remaining 376 patients (48.2%) had an EF of 50% or below, indicative of HFrEF or HFmrEF (Fig. **1**).

Among the 376 patients with HFrEF and HFmrEF, only 97 (26%) underwent screening for ID. Of those screened, 52 patients (54%) were found to have ID. Only 10 patients with ID (19%) received IV FCM, of whom only 8 patients (15%) received the full recommended dose of 1000 mg IV FCM. Anemia was present in about 87% of the patients. The screening rate for ID was much higher among patients with anemia (33.9%; 83/245) compared to those without anemia (10.7%; 14/131), *p* < 0.001.

A comparison of HF patients with ID [n=52] and those without ID [n=45] revealed no statistically significant differences in the median age [62.75 *vs*. 68 years, *p* value 0.^22^], male: female ratio [1:1.6 *vs*. 1:1.^36^], or prevalence of co-morbidities such as diabetes [69% *vs*. 64%, *p* value 0.311], IHD [73% *vs*. 60%, p=0.08772], PVD [15% *vs.* 13%, p=0.38857], or CKD [69%, p=0.03595], Table **1**.

## 4 DISCUSSION

Over the last decade, IV FCM has emerged as an important part of GDMT for HF. ID and anemia are dynamic processes, and patients with HF who are found to be replete in iron at the time of admission can develop ID later. Graham *et al*. also reported increased all-cause mortality in HF patients with persistent ID compared to those who never had ID. This suggests the importance of regular screening for ID in HF at regular intervals [12]. Apart from a recently published randomized controlled trial, HEART-FID [31], the majority of clinical trials support the use of IV FCM in acute and chronic HF patients. The 2022 AHA guidelines gave a class 2a indication for FCM in HFrEF, whereas the recent ESC guidelines have upgraded IV FCM to a class 1 indication. In fact, FCM is the only class 1–indicated therapy for improving exercise capacity in HF according to major guidelines [10, 32]. This study provides valuable insights into the prevalence, screening patterns, and therapeutic interventions for ID in HF patients in Oman, offering important regional data.

Among our study population, the screening rate of ID was quite low, with only 26% of 376 patients being screened according to the guidelines. Of those screened, about half were found to have ID as per the FAIR-HF definition of ID. The screening rate observed in this study, while notably low, is comparable to international reports [33, 34]. The Swedish HF registry showed an improvement in screening rates over time; however, it remained below 25% in 2018 [35, 36]. Similarly, a study from Toronto reported a screening rate for ID of 36.3% among anemic heart failure patients [37]. In England, an analysis of hospital episode data from 2013 to 2016 found that only 14.2% of hospital admissions for HF had a secondary diagnosis of ID or iron deficiency anemia (IDA) established [38].

We observed a statistically significant difference in the ID screening rates between anemic and non-anemic HF patients (34% *vs*. 10.7%, *p* < 0.001). This disparity highlights a potential bias in clinical practice, where anemia often serves as the primary trigger for investigating ID. The updated ESC and AHA guidelines emphasize the importance of screening for ID in all HF patients, regardless of anemia status, due to its established impact on functional capacity, quality of life, and clinical outcomes [10-12].

The reasons for low screening rates for ID among HF patients are usually multifactorial. Kido and colleagues identified lack of access to health care, lack of knowledge among healthcare providers, concerns about potential side effects, and the cost of IV therapy as the main challenges in the US [4]. Infections are a common precipitant of acute HF exacerbations, reported in approximately 38% of hospitalized acute HF patients, and are associated with increased morbidity and mortality [39, 40]. Studies have shown that concerns about the safety of IV iron therapy during an acute infective illness can lead to a reduction in the rate of IV FCM administration [4]. A recent meta-analysis published in 2021 showed no significant risk of increased morbidity or length of stay after IV iron therapy during an infection [41]. Similarly, in a major randomized controlled trial (IRON MAN), the authors found no evidence of increased risk of hospitalization or mortality from infection after IV FCM therapy. Out of 569 patients treated with FCM over a duration of approximately 5 years, only one allergic reaction was reported. This study signifies the safety of IV iron therapy in all hospitalized HF patients, regardless of their infection status [15].

In our study, therapeutic interventions for patients with HF and ID revealed significant gaps in the standard of care. The majority of the patients identified as ID did not receive any IV iron replacement. Similar findings were reported in a previous longitudinal cohort study from Spain involving 711 HF patients. ID was identified in 37.9% of visits, yet IV iron was administered in only 7.3% of visits, with 32.8% of patients receiving treatment during follow-up [42]. A quality improvement cohort study from Toronto reported that only 17.2% of patients received IV iron, despite 64.4% being diagnosed with IDA [17].

In summary, the clinical practice for the diagnosis and management of ID in HF within our healthcare institution exhibits only minor distinctions when compared to practices in other countries. Nevertheless, there exists significant potential for enhancement in the management of ID among HF patients, both in Oman and worldwide. Following an extensive review of published data and the most recent guidelines, Kido and colleagues disseminated a comprehensive framework for intravenous iron therapy applicable to both inpatient and outpatient settings in 2024. They recommended the implementation of standardized pathways to ensure adherence, foster awareness among healthcare practitioners, and mitigate logistical challenges [4]. This structured framework may be employed in its entirety or in a modified version within Oman to achieve target goals in GDMT for patients diagnosed with HFrEF and HFmrEF.

## CONCLUSION

The majority of hospitalized HF patients did not undergo screening for ID, despite strong recommendations by the AHA and ESC guidelines. Among those who were diagnosed with ID, treatment with the appropriate dose of IV FCM remained quite low. These findings highlight a significant gap in GDMT for HF patients. Addressing these gaps through standardized protocols and educational interventions can enhance the early detection and effective management of ID.

## LIMITATIONS AND RECOMMENDATIONS

This was a single-center retrospective study; therefore, the results cannot be extrapolated to other healthcare centers in Oman or in the Middle East. The long-term outcomes of IV FCM, such as length of hospital stay, re-hospitalization rate, improvement in quality of life, and all-cause mortality, cannot be assessed due to the lack of outcome data in this study. However, these parameters were beyond the scope of this study, as the aim was to analyze the prevalence of ID and adherence to guideline-directed therapy for ID in HF patients. The prevalence of ID in all HF patients cannot be precisely evaluated due to the low screening rate. In addition, the precise timing of IV FCM administration during the patients' hospital stay could not be identified due to the lack of time stamps on the data sheet derived from electronic resources.

We recommend further studies to analyze the barriers to implementing guideline-directed IV iron therapy in HF patients in Oman, propose strategies for improvement, and ensure adherence to these strategies.

## AUTHORS’ CONTRIBUTIONS

The authors confirm their contributions to the paper as follows: Study conception and design: M.S., K.A.H.; Data collection: Y.A.S., A.A.N., M.A.S., A.A.J., Y.A.A., A.A.M.; Analysis and interpretation of results: M.S., K.A.H., A.A.A., K.A.Z., A.A.R.; Draft manuscript: M.S., K.A.H., S.A.B., S.A.J. All authors reviewed the results and approved the final version of the manuscript.

## Figures and Tables

**Fig. (1):**
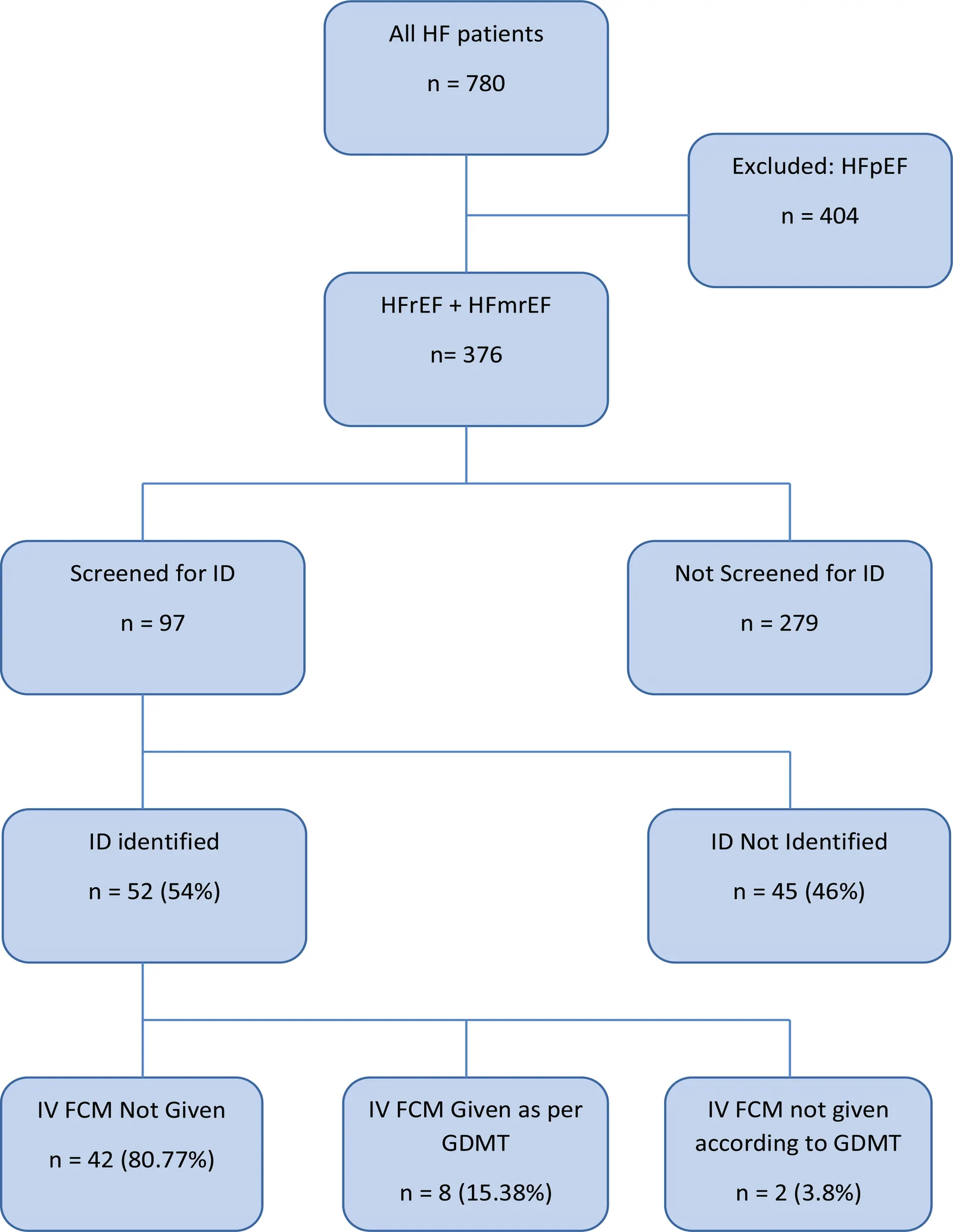
Flow chart of patient selection, screening, and treatment for ID in HFrEF and HFmrEF. **Abbreviations:** HF; heart failure, HFrEF; heart failure with reduced ejection fraction, HFmrEF; heart failure with mildly reduced ejection fraction, HFpEF; heart failure with preserved ejection fraction, ID; iron deficiency, IV; intravenous, FCM; Ferric carboxymaltose, GDMT; guideline-directed medical therapy.

**Table 1:** Comparison of demographics and comorbidities in HFrEF and HFmrEF patients with ID and those without ID.

Characteristic	Total [N = 97]	HFrEF and HFmrEF patients with ID [N = 52]	HFrEF and HFmrEF Patients without ID [N = 45]	*p-*value
Sex				
Male N [%]	39 [40.21%]	20 [38.46%]	19 [42.22%]	0.706
Female N [%]	58 [59.79%]	32 [61.54%]	26 [57.78%]	-
Age in years [Median (IQR)]	68 [61–78]	68.5 [62.5–80.5]	68 [60–77]	0.26
LVEF% % [Median (IQR)]	35 [27–41]	0.341 [25–41]	35 [28.5–41]	0.671
Hb in g/L [Median (IQR)]	9.9 [8.5–11.5]	10 [8.8–12.05]	9.1 [8.2–11]	0.045
Creatinine µmol/L [Median (IQR)]	135 [90–207]	126.5 [76–158]	167 [100–282]	0.011
Diabetes N [%]	65 [67.01%]	36 [69.23%]	29 [64.44%]	0.617
CKD N [%]	67 [69.07%]	36 [69.23%]	31 [68.89%]	0.971
IHD N [%]	65 [67.01%]	38 [73.08%]	27 [60.00%]	0.172
PVD N [%]	14 [14.43%]	8 [15.38%]	6 [13.33%]	0.774
Stroke N [%]	21 [21.65%]	8 [15.38%]	13 [28.89%]	0.107
Smoking N [%]	14 [14.43%]	9 [17.31%]	5 [11.11%]	0.386

**Abbreviations:** HF; heart failure, HFrEF; heart failure with reduced ejection fraction, HFmrEF; heart failure with mildly reduced ejection fraction, ID; iron deficiency, LVEF; left ventricular ejection fraction, Hb: hemoglobin, CKD; chronic kidney disease, IHD; ischemic heart disease, PVD; peripheral vascular disease.

## Data Availability

The authors confirm that the data supporting the findings of this research are available within the article.

## STANDARDS OF REPORTING

STROBE guidelines were followed.
